# Large language models in clinical nutrition: an overview of its applications, capabilities, limitations, and potential future prospects

**DOI:** 10.3389/fnut.2025.1635682

**Published:** 2025-08-07

**Authors:** Jamal Belkhouribchia, Joeri Jan Pen

**Affiliations:** ^1^Endocrinology Center Hasselt, Hasselt, Belgium; ^2^Department of Nutrition, UZ Brussel, Vrije Universiteit Brussel (VUB), Brussels, Belgium

**Keywords:** large language models, clinical nutrition, artificial intelligence, personalized nutrition therapy, personalized dietary recommendations, retrieval-augmented generation large language models

## Abstract

The integration of large language models (LLMs) into clinical nutrition marks a transformative advancement, offering promising solutions for enhancing patient care, personalizing dietary recommendations, and supporting evidence-based clinical decision-making. Trained on extensive text corpora and powered by transformer-based architectures, LLMs demonstrate remarkable capabilities in natural language understanding and generation. This review provides an overview of their current and potential applications in clinical nutrition, focusing on key technologies including prompt engineering, fine-tuning, retrieval-augmented generation, and multimodal integration. These enhancements increase domain relevance, factual accuracy, and contextual responsiveness, enabling LLMs to deliver more reliable outputs in nutrition-related tasks. Recent studies have shown LLMs’ utility in dietary planning, nutritional education, obesity management, and malnutrition risk assessment. Despite these advances, challenges remain. Limitations in reasoning, factual accuracy, and domain specificity, along with risks of bias and hallucination, underscore the need for rigorous validation and human oversight. Furthermore, ethical considerations, environmental costs, and infrastructural integration must be addressed before widespread adoption. Future directions include combining LLMs with predictive analytics, integrating them with electronic health records and wearables, and adapting them for multilingual, culturally sensitive dietary guidance. LLMs also hold potential as research and educational tools, assisting in literature synthesis and patient engagement. Their transformative promise depends on cross-disciplinary collaboration, responsible deployment, and clinician training. Ultimately, while LLMs are not a replacement for healthcare professionals, they offer powerful augmentation tools for delivering scalable, personalized, and data-driven nutritional care in an increasingly complex healthcare environment.

## 1 Introduction

The field of clinical nutrition is facing a transformative change with the advent of large language models (LLMs), a domain within artificial intelligence (AI). These advanced systems are trained on vast datasets and exhibit remarkable generative capabilities (1–8). LLMs can produce diverse content autonomously, including interpreting complex queries, synthesizing information, and providing human-like responses (9–11). In clinical nutrition, where decision-making often involves integrating patient-specific data, scientific evidence, and evolving guidelines, the potential of LLMs is profound (1, 12). They promise to streamline workflows, enhance personalized care, and support clinicians in making data-driven decisions. However, while their capabilities are impressive, understanding their role, limitations, and ethical considerations is essential for responsible integration into clinical practice (2, 13–15).

Clinical nutrition involves screening, diagnosing, treating, and monitoring patients with specific nutritional issues or diseases that require dietary adjustments. To support this process, nutrition specialists rely on medical records, anthropometric measurements, laboratory results, and dietary information to develop personalized nutritional plans that align with current scientific guidelines. LLMs can significantly enhance and streamline this workflow by analyzing patient data, incorporating evidence-based guidelines, and assisting physicians or dietitians in the diagnosis and management of nutritional problems.

Although LLMs and LLM-based tools such as ChatGPT (Generative Pretrained Transformer) are widely adopted across various industries, their potential and application within clinical nutrition remain largely unexplored. Before these technologies can be integrated into routine practice, it is essential that nutrition specialists gain a thorough understanding of their underlying mechanisms, capabilities, and limitations. Furthermore, LLMs must operate transparently and provide users with the ability to verify the sources upon which their recommendations are based.

This article explores how LLMs are shaping the future of clinical nutrition, offering insights into their applications, benefits, challenges, and the potential to revolutionize patient care.

## 2 Natural language processing

Natural Language Processing (NLP) is a multidisciplinary field at the intersection of linguistics, computer science, and artificial intelligence, aiming to enable machines to understand, process, and generate human language in a meaningful way. NLP applications rely on diverse methodologies, ranging from traditional rule-based systems to cutting-edge machine learning techniques (16). Within the broader field of NLP, LLMs have emerged as a transformative technology. They represent a specialized class of machine learning models designed to handle complex language tasks by leveraging vast amounts of pretraining data (17–19).

Building on their general capabilities, LLMs enhance the functionality of NLP systems by allowing more accurate interpretation of complex language, including specialized terminology and context-dependent meaning. When applied to clinical domains, these models can be adapted to handle discipline-specific content with a high degree of relevance. In clinical nutrition, this opens the possibility to efficiently process and interpret diverse sources of textual information, such as dietary records, medical notes, and scientific publications, supporting clinicians in translating data into meaningful, individualized nutritional advice.

## 3 LLM types and architecture

Understanding the types and underlying architectures of LLMs is crucial to appreciating how they process and generate language in clinical nutrition applications. LLMs are built on a type of deep learning architecture known as the transformer, which has become foundational in NLP (20). Transformers process language by dividing text into units called tokens. Tokens may represent words, parts of words, or characters. Tokens are then converted into numerical representations, allowing the model to analyze relationships and contextual meaning (21–23). A key feature of Transformer-based models is their attention mechanism, which enables them to weigh the relevance of different words in a sentence or paragraph, even if they are far apart. This mechanism is what allows LLMs to interpret nuanced queries and maintain contextual coherence over long passages of text (24–26). Transformer models generally fall into three categories based on how they process information: encoder-only, decoder-only, and encoder-decoder models (27, 28).

Encoder-only models are designed to understand and analyze input text (29). They process text bidirectionally, considering both what comes before and after a given token, which makes them particularly effective for tasks like information extraction, classification, or identifying relevant clinical features in unstructured data.

Decoder-only models, such as GPT (Generative Pre-trained Transformer), are optimized for generating text (30). They process text in a unidirectional manner, predicting the next token based on the previous ones. These models excel at generating coherent, human-like responses and are well suited for use cases like clinical documentation support, patient education, or answering open-ended questions.

Encoder-decoder models are designed to take in an input via the encoder, transform it into an internal representation, and then generate a corresponding output via the decoder. This structure is particularly useful for tasks like summarization, translation, or structured question-answering, where the model must fully understand the input and produce a targeted response (31).

Each architecture has strengths depending on the intended use (32). In clinical contexts such as nutrition, selecting the appropriate type of large language model is essential for optimizing outcomes. Whether the task involves analysis, content generation, or structured interaction, matching the model to the use case is critical.

A clear understanding of LLMs’ architectural distinctions is critical to aligning model capabilities with specific clinical tasks and ensuring meaningful, reliable outcomes in nutritional practice.

## 4 Techniques to enhance LLMs

Although LLMs are typically pre-trained on broad, general-purpose data, several methods can improve their performance, accuracy, and domain relevance, particularly for specialized applications in fields like clinical nutrition. Key enhancement techniques include prompt engineering, fine-tuning, retrieval-augmented generation (RAG), and multimodal integration (33–36).

### 4.1 Prompt engineering

Prompt engineering is the practice of structuring user inputs in a way that elicits more precise, relevant, or task-specific outputs from the model (37, 38). Because LLMs are highly sensitive to the phrasing and context of a prompt, small adjustments can significantly influence the quality of the response (39). For instance, a general query like “What is the dietary treatment for diabetes?” may yield vague or generic output. Rephrasing it as “Provide nutritional treatment for type 2 diabetes in adults, based on current clinical guidelines, and include references” tends to produce more structured and clinically relevant results. Several strategies exist. Zero-shot prompting asks the model to perform a task without prior examples, relying on its general training. Few-shot prompting provides illustrative examples within the prompt to guide the model’s behavior (40). Chain-of-thought prompting instructs the model to reason step-by-step, thereby improving its performance on complex or multi-step queries (41). Prompt engineering is particularly valuable when model retraining is not feasible. It allows clinicians to adapt general-purpose models for specific tasks using careful phrasing, without altering the model itself.

### 4.2 Fine-tuning

Fine-tuning involves updating a pre-trained model using additional data specific to a task, institution, or clinical domain (42). This process refines the model’s internal representations, improving performance on highly specialized queries. Full fine-tuning adjusts all model parameters and is effective but computationally intensive. It also carries a risk of overfitting when the dataset is small. Parameter-efficient methods, such as Low-Rank Adaptation (LoRA), modify only a subset of parameters, reducing resource requirements while preserving general capabilities (43). Domain adaptation, a subset of fine-tuning, uses field-specific datasets (e.g., clinical nutrition guidelines) to align the model with professional language, knowledge, and priorities in that domain. In practice, fine-tuned models can more reliably answer domain-specific questions, generate summaries from patient records, or support documentation using accurate terminology.

### 4.3 Retrieval-augmented generation large language models

Retrieval-augmented generation large language models (RAG-LLMs) combine a language model with an external retrieval mechanism that supplies relevant documents or facts in real time, thereby increasing factual accuracy and contextual relevance (44, 45). This approach addresses a key limitation of LLMs: their reliance on static training data, which can become outdated or incomplete. In a RAG-LLM setup, when a user submits a query, the system first retrieves up-to-date or domain-specific documents, such as recent guidelines or clinical studies, and then provides this context to the LLM. The model uses this information to generate an informed, citation-backed response. RAG-LLMs are especially valuable in clinical domains like nutrition, where evidence changes regularly and accuracy is essential. RAG-LLMs enable dynamic access to trusted knowledge sources, improving factual consistency and clinical relevance.

### 4.4 Multimodal integration

Multimodal LLMs extend traditional text-based models by incorporating other forms of input, such as images or audio (46–48). This opens the door to richer, context-aware outputs across more complex workflows. In clinical nutrition, potential use cases include: interpreting food photos to assess dietary intake; combining blood test results and anthropometric data with textual dietary advice; supporting visually enhanced patient education materials. Although still emerging, multimodal models represent the next stage in LLM development, especially in domains where information comes in diverse formats. The use of multimodel LLMs in clinical nutrition has yet to commence, there are, however, examples in other fields of medicine (49).

In summary, enhancement techniques such as prompt engineering, fine-tuning, RAG, and multimodal integration significantly improve the practical utility of LLMs in clinical nutritional. By making models more responsive, accurate, and context-aware, these methods allow LLMs to meet the demands of specialized domains like clinical nutrition while maintaining clinical reliability.

## 5 LLM applications in clinical nutrition

**Figure 1:**
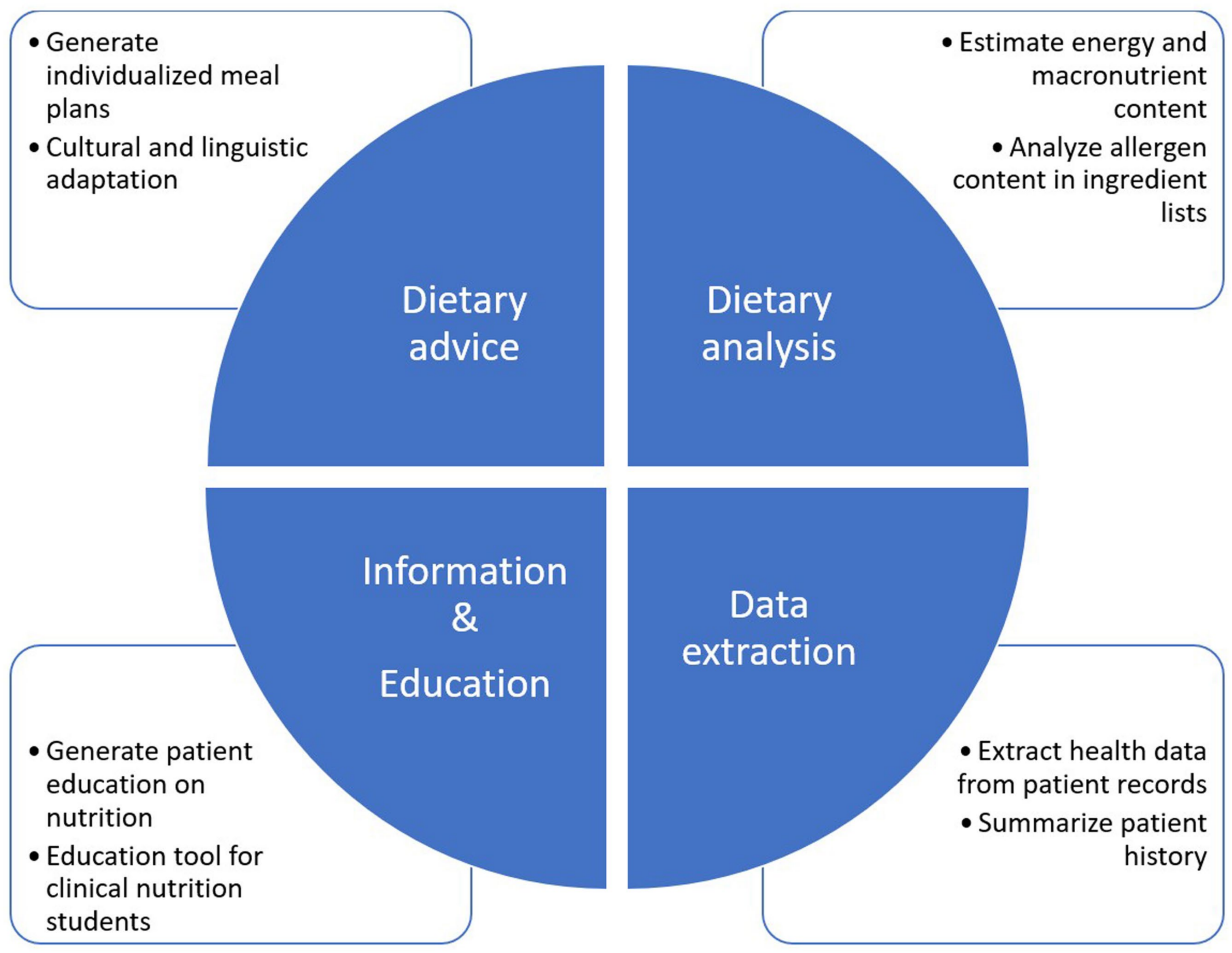
Overview of the possibilities of large language models in clinical nutrition.

The application of LLMs in clinical nutrition is expanding rapidly, with increasing adoption and diverse use cases, see Figure 1. This section highlights several key examples to illustrate their potential impact and also their shortcomings. A summary is given in Table 1.

**Table 1:** Overview of some LLM application examples that are used in clinical nutrition.

Application type	Author and year	Objectives	Findings	Limitations
Dietary recommendations	Singh B et al. 2023 (50)	Meta-analysis: Chatbot to improve physical activity, diet end sleep	Improved physical activity, diet, and sleep; AI-driven chatbots are more effective than voice-based	Low study quality studies and heterogeneity among interventions
	Arslan S. 2023 (51)	ChatGPT for personalized diet in obesity and prediction of obesity related diseases	ChatGPT offers personalized support in obesity management	Contextual limitations, lack of emotional intelligence, privacy and ethical concerns
	Haman M et al. 2024 (52)	ChatGPT for dietary planning and weight management	High accuracy in energy estimates; consistent nutrient predictions	Inaccuracy for specific nutrients; inability to account for chronic health conditions; generation of plausible yet inaccurate information
	Khan U. 2024 (53)	ChatGPT for personalized diet in protein-energy malnutrition	Provides personalized advice and monitoring for protein-energy malnutrition	Lacks ability of physical assessment; relies on input quality; not suited for complex cases
	Wang LC et al. 2024 (54)	ChatGPT for personalized nutrition in dialysis patients	Good recipe generation; poor nutrient analysis	Underestimated key nutrients, medical applicability was limited
	Adilmetova G et al. 2024 (55)	ChatGPT for personalized diet in English, Kazakh, and Russian	Effective in English/Russian, ineffective in Kazakh	Poor performance in underrepresented languages
	Hieronimus B et al. 2024 (56)	ChatGPT and Bard for dietary recommendations in omnivorous and restricted diets	Plans often nutritionally inadequate, B12 often lacking	Not suitable for restrictive diets
	Niszczota P et al. 2023 (57)	ChatGPT for dietary recommendations for patients with food allergies	Correctly excluded allergens in most cases	Critical errors, monotonous menus
	Papastratis I et al. 2024 (58)	ChatGPT + deep generative networks for personalized meal plans	More accurate and explainable than ChatGPT	Still needs more dietary diversity and user feedback
Information and education	Barlas T et al. 2024 (59)	ChatGPT for assessment and management of obesity in type 2 diabetes	Good assessment accuracy; weaker in therapy	Outdated info, lack of guideline citations
	Kirk D et al. 2023 (61)	ChatGPT for answering common nutrition questions	ChatGPT outperformed dietitians on 5 of 8 questions	Potential overconfidence in AI answers
	Liao LL et al. 2024 (62)	ChatGPT for educational purposes	High nutrition literacy, poor completeness and depth	Impractical advice, lacks rigor
Meal analysis	Hoang YN et al. 2023 (63)	ChatGPT for estimating the energy and macronutrient content of food items	Energy and macronutrients fairly accurate; protein overestimated	Lacks personalization, poor portion size handling
	Sun et al. 2023 (64)	ChatGPT + Dino V2 for diet recommendation and ingredient analysis in type 2 diabetes	Passed dietitian exam; high image recognition accuracy	variability in responses and inconsistencies in local food responses
Data extraction	Alkhalaf M et al. 2024 (65)	Llama2 with and without RAG for extracting malnutrition related data from electronic health records	RAG improved summarization and risk factor extraction	Hallucinations when information is missing or implicit

AI, Artificial Intelligence; DINO, self-DIstillation with NO labels; GPT, Generative Pretrained Transformer; RAG, Retrieval Augmented Generation.

This review does not aim for an exhaustive coverage but instead provides a curated overview of illustrative examples to highlight the range of current and emerging applications of LLMs in clinical nutrition. Articles were selected based on their relevance, recency (2023–2025), and ability to demonstrate specific clinical or technological use cases. Selection was guided by the authors’ expertise, supplemented by targeted searches in PubMed and Google Scholar using keywords such as “ChatGPT,” “large language models,” “clinical nutrition,” and “personalized nutrition.” Studies were grouped thematically into five domains – dietary recommendations, information and education, ingredient analysis, data extraction, and cross-disciplinary innovations – to reflect common patterns and areas of interest. This approach prioritizes breadth and relevance over completeness, aiming to inform and to inspire future research and implementation.

### 5.1 LLMs for dietary recommendations

Singh and colleagues performed a meta-analysis looking at chatbot interventions designed to improve physical activity, diet and sleep (50). Analyzing 19 trials with sample sizes ranging from 25 to 958 and participant ages between 9 and 71, the study found significant improvements in physical activity, daily steps, moderate-to-vigorous physical activity, fruit and vegetable consumption, sleep duration, and sleep quality. Text-based and AI-driven chatbots outperformed voice chatbots in dietary improvements, and multicomponent interventions were more effective than chatbot-only approaches for enhancing sleep outcomes. Despite a predominance of low-quality studies, findings demonstrate that chatbot interventions are effective across diverse populations and settings (Table 2).

**Table 2:** Glossary of technical terminology.

Terminology	Explanation
Attention mechanism	The attention mechanism is a feature in language models that helps them determine which words in a sentence are most important when interpreting meaning. For example, in the phrase “she gave him water because he was thirsty,” attention helps the model understand that “he” refers to the one who is thirsty. This allows more accurate and context-aware responses.
Embedding	Embedding refers to converting words or phrases into numerical codes (vectors) that a computer can process. These codes preserve the meaning and relationships between words (e.g., “apple” and “fruit” are close together), helping the model understand context and semantics.
Encoder-only / Decoder-only / Encoder-decoder models	These are types of language models. Encoder-only models analyze and understand text (useful for identifying medical terms in a record), decoder-only models generate new text (e.g., writing summaries), and encoder-decoder models do both (e.g., translating a diet note into lay language). The model type is chosen based on the task’s needs.
Fine-tuning	Fine-tuning means adjusting a pre-trained language model using a smaller set of specialized data, such as clinical nutrition guidelines. This improves the model’s accuracy for specific tasks or domains by aligning it with expert language and knowledge in that field.
Generative Pretrained Transformer (GPT)	GPT is a specific type of large language model designed to generate human-like text. It can answer questions, write explanations, or summarize content based on prior training on a vast range of internet and literature sources.
Hallucinations	Hallucinations occur when an AI model produces convincing but incorrect or fabricated information. For example, it may invent a study or cite a nonexistent guideline. This is a known risk when using language models in clinical settings.
Large Language Models (LLMs)	LLMs are advanced AI tools trained on vast amounts of text data to understand and generate human-like language. They can assist in clinical tasks such as summarizing patient notes, answering nutrition questions, or drafting educational materials.
Loss function	The loss function measures how far off the model’s predictions are from the correct answers during training. It helps guide improvements in the model’s performance by minimizing errors over time.
Low-Rank Adaptation (LoRA)	LoRA is a resource-efficient method of fine-tuning that updates only part of a model instead of all its components. This allows customization of models for specific tasks without the need of powerful hardware or massive datasets.
Natural Language Processing (NLP)	NLP is the field of artificial intelligence that enables machines to understand and use human language. LLMs are a specific type of machine learning within the broader field of NLP.
Opaque / Black-box models	These refer to artificial intelligence systems where the reasoning behind an output is unclear or difficult to trace. In clinical practice, this might raise concerns about trust, transparency, and accountability when using such tools for patient care.
Prompt engineering	Prompt engineering involves carefully wording a question or instruction to get the best possible response from a language model. For example, asking “List dietary recommendations for type 2 diabetes in adults” will yield more useful results than simply typing “diabetes diet.”
Recurrent Neural Networks (RNNs)	RNNs are an older type of AI model designed to process sequences of data, like sentences or time-series information. They have mostly been replaced by transformer-based models due to limitations in handling long or complex text.
Retrieval-Augmented Generation (RAG)	RAG is a method that improves accuracy of LLMs by providing a knowledge base to the LLM in which answers for queries can be retrieved, rather than relying only on what the model was originally trained on. This allows for flexibility and the possibility to update the knowledge base.
Tokens and tokenization	Tokenization is the process of breaking down text into smaller units (tokens), such as words or subwords, so the model can analyze them. For example, “high-protein diet” might be split into “high,” “-protein,” and “diet” for the model to process accurately.
Transformer-based architectures	Transformers are the foundation of most modern language models. They allow the model to understand the meaning of words based on their context in a sentence, enabling more accurate interpretation and generation of complex medical or dietary language.
Variational Autoencoders (VAEs)	VAEs are AI models that can learn patterns from data and generate new, similar data—such as customized meal plans. They are often used for tasks like modeling patient profiles or generating personalized health content.
Zero-shot prompting / Few-shot prompting / Chain-of-thought prompting	These are techniques for guiding model responses. Zero-shot prompting gives no examples, few-shot gives a few examples, and chain-of-thought encourages step-by-step reasoning. These strategies help improve accuracy when using general-purpose language models in clinical tasks.

Arslan explored the potential of ChatGPT, an AI-driven language model, in the treatment of obesity, a growing global health concern (51). ChatGPT’s capabilities include providing personalized recommendations for nutrition plans, exercise programs, and psychological support, as well as developing predictive models for obesity-related diseases like diabetes and cardiovascular conditions. These features could enhance weight management and reduce associated health risks through tailored and adaptive treatment strategies. However, the study highlights challenges such as the model’s limited contextual understanding, lack of emotional intelligence, privacy and security concerns, and ethical considerations regarding accountability for AI-generated advice. Despite these limitations, ChatGPT presents promising opportunities in obesity management, though its application in healthcare requires cautious implementation and further research.

Haman et al. evaluated the accuracy and reliability of ChatGPT in generating nutritional information for dietary planning and weight management (52). Utilizing the United States Department of Agriculture (USDA) Food Data Central as a reference, ChatGPT demonstrated high accuracy in estimating energy values, with 97% of its predictions falling within a 40% margin of USDA data. The model exhibited consistency across nutrient estimates, as indicated by low coefficients of variation, and effectively generated daily meal plans, with all meals adhering to a 30% margin of USDA caloric values. However, limitations were observed, including variable accuracy for specific nutrients, the inability to account for chronic health conditions, and the potential for generating plausible yet inaccurate information. While ChatGPT showed promise as a supplementary tool, the study emphasized ChatGPT should not replace professional medical or dietary guidance.

Khan looked at the potential of ChatGPT in addressing protein-energy malnutrition (PEM), a critical global health issue (53). ChatGPT demonstrates the ability to provide personalized dietary recommendations, guidance on protein-rich food choices, psychological support, and real-time monitoring to improve PEM interventions. It can also analyze PEM-related data to inform research and policymaking. However, limitations such as the inability to perform physical assessments, reliance on user inputs, susceptibility to bias, and inadequate handling of complex cases highlight the importance of integrating AI tools with healthcare professionals. Collaborative efforts combining AI capabilities and human expertise are essential for achieving accurate diagnoses, individualized treatment plans, and comprehensive care in PEM management.

Wang and co-workers explored ChatGPT-4’s ability to support personalized nutritional advice for dialysis patients by generating meal plans based on virtual patient profiles created via Monte Carlo simulation (54). A renal dietitian evaluated the generated recipes, cooking instructions, and nutritional analyses, rating the instructions highly (5/5) but the recipes and nutritional analysis lower (3/5 and 2/5, respectively). ChatGPT’s nutritional analysis underestimated key nutrients, including calories (36%), protein (28%), and potassium (49%), among others. Recipe translations into multiple languages were rated as reliable (4/5). While ChatGPT-4 demonstrates potential for personalized guidance, significant improvements are needed for accurate nutritional analysis and medical applicability. Although this study states that translations in different languages were reliable, this is not always the case when using LLMs in clinical nutrition. Adilmetova et al. evaluated ChatGPT-4’s ability to provide personalized, evidence-based dietary recommendations in English, Kazakh, and Russian in Central Asia using 50 mock patient profiles (55). Performance was assessed for personalization, consistency, and practicality, revealing moderate effectiveness in English and Russian but unsuitability for Kazakh due to insufficient outputs. Statistically significant differences (*p* < 0.001) were observed across the three languages, with English and Russian outperforming Kazakh. The findings highlight ChatGPT-4’s limitations in underrepresented languages, emphasizing the need for customized models tailored to local diets and sociocultural contexts.

Using popular LLMs like ChatGPT in clinical practice may seem appealing. However, despite their potential utility, they carry the risk of generating inaccurate or misleading information. Hieronimus and colleagues assessed the ability of AI chatbots ChatGPT and Bard (now Gemini) to generate meal plans meeting dietary reference intakes (DRIs) for omnivorous, vegetarian, and vegan diets (56). Across 108 meal plans, nutrient analysis showed lower energy and carbohydrate content but excess protein relative to DRIs. Common deficiencies included vitamin D and fluoride, with vegan plans also lacking vitamin B12. ChatGPT suggested B12 supplementation in some cases, while Bard did not. No significant differences were observed between the chatbots or prompts. While these tools provide general dietary inspiration, they are unsuitable for creating nutritionally adequate plans, particularly for restrictive diets.

Niszczota and Rybicka evaluated ChatGPT’s ability to create elimination diets for individuals with food allergies (57). They focused on safety, accuracy, and variety. While ChatGPT correctly excluded allergens in most cases, critical errors were identified, such as including allergenic ingredients like almond milk in nut-free diets. The model also demonstrated inaccuracies in energy and portion calculations and generated monotonous menus with limited variety. Despite these shortcomings, ChatGPT adheres to some basic dietary guidelines and shows potential for improving accessibility to dietary advice. However, the study highlights the risks of using ChatGPT for critical health tasks, emphasizing the need for model fine-tuning and further research to enhance safety and reliability in nutritional recommendations.

In order to improve LLMs performance, more sophisticated technologies can be incorporated. Papastratis et al. introduce a novel AI-based diet recommendation system that combines deep generative networks, such as variational autoencoders and recurrent neural networks, with LLMs like ChatGPT to provide accurate, personalized weekly meal plans (58). By modeling user profiles (e.g., anthropometric measurements and medical conditions) and embedding predefined nutritional guidelines from EFSA (European Food Safety Authority) and the WHO (World Health Organization) as loss functions, the system ensures outputs that align with expert-validated dietary standards while maintaining high accuracy and explainability. The integration of ChatGPT expands the meal database by generating additional meal options from diverse cuisines, enhancing variety and applicability across different populations without acting as a retrieval system. Evaluations on 3,000 virtual and 1,000 real user profiles demonstrated superior performance in energy and macronutrient alignment compared to ChatGPT, showcasing its precision and potential for seamless integration into healthcare and fitness applications. Future work will focus on accommodating more dietary preferences, international cuisines, and real-world user feedback to further refine its effectiveness.

### 5.2 LLMs for information and education

Barlas and colleagues assessed the credibility of ChatGPT-3.5 in providing information on the assessment and management of obesity in type 2 diabetes (T2D) based on the latest American Diabetes Association (ADA) and American Association of Clinical Endocrinology (AACE) guidelines (59). In a cross-sectional design, 20 patient-focused questions were posed by experienced endocrinologists, and responses were categorized as compatible, compatible but insufficient, partially incompatible, or incompatible with the guidelines. ChatGPT demonstrated 100% compatibility in the assessment of obesity but lower adherence in therapy-related sections, including nutrition, pharmacotherapy, and surgical interventions, often requiring additional prompts for completeness. While ChatGPT provided clear, systematic, and understandable answers, it lacked currency regarding recently updated information and specificity in sourcing guidelines. These findings emphasize that although ChatGPT holds potential as a supplementary tool for information retrieval, it should not replace healthcare professionals’ patient-centered approaches, as individualized care and human oversight remain critical for ensuring accuracy and reliability in medical guidance.

Although LLMs promise to greatly enhance the outcome of patients in clinical nutrition, adoption by patients could pose a challenge. Vandelanotte et al. explored user perceptions and expectations of an artificially intelligent physical activity digital assistant through six focus groups that consisted of 45 participants (60). Participants expressed enthusiasm for such an assistant, emphasizing the importance of customizable features, including notifications, personality, and appearance. While participants were open to sharing information for personalization, their willingness varied significantly. Despite privacy concerns, they supported the use of AI and machine learning for enhanced functionality. However, the strong demand for personalization presents challenges in terms of development cost and complexity, highlighting the need for careful design to meet user expectations.

LLMs like ChatGPT can, however, provide useful answers to general nutrition-related questions. Kirk et al. evaluated ChatGPT’s competency in answering common nutrition questions compared to dieticians’ responses (61). Questions and answers were graded by experts on scientific correctness, actionability, and comprehensibility. ChatGPT outperformed dieticians in overall scores for five out of eight questions, excelling in scientific correctness, actionability, and comprehensibility in several instances. Dieticians’ answers did not surpass ChatGPT’s scores in any category. These findings suggest that ChatGPT can effectively address frequently asked nutrition questions, highlighting its potential as a supportive tool for providing nutrition information.

LLMs are increasingly used as a tool in clinical nutrition practice; they are also being employed as an educational tool. Liao and colleagues evaluated ChatGPT’s performance in providing dietary advice to college students, assessed by 30 dietitians and a nutrition literacy (NL) test (62). While ChatGPT demonstrated high accuracy in the NL test (84.38%), surpassing the NL level of Taiwanese students, its responses were often incomplete, impractical, and lacked thoroughness, raising concerns about potential misunderstandings. Dietitians frequently cited a lack of rigor in the information provided. Despite these gaps, ChatGPT’s readability and potential as a supplementary educational tool were recognized, emphasizing the need for improved AI guidelines and training materials to enhance its effectiveness in nutrition education.

### 5.3 LLMs for ingredient analysis

LLMs can also be used to estimate energy and macronutrient content of food. However, their performance is still suboptimal. Hoang and co-workers evaluated the reliability of ChatGPT-3.5 and ChatGPT-4 in estimating the energy and macronutrient content of 222 food items across eight menus, comparing their results to nutritionists’ recommendations (63). While AI estimations for energy, carbohydrates, and fats were consistent with nutritionists’ data, protein estimates showed significant discrepancies. ChatGPT-4 outperformed ChatGPT-3.5 in accuracy but overestimated protein content. Both chatbots provided accurate energy estimates within ±10% for 35–48% of food items. Despite these limitations, the study highlights AI chatbots as convenient tools for basic nutritional analysis but notes their inability to offer personalized dietary advice or account for household portion sizes. Enhancements in AI specialization for nutrition could significantly improve their utility in dietetics.

Sun et al. examined an AI-based nutritionist program designed to address the challenges of nutritional management in patients with type 2 diabetes mellitus in China (64). The program integrates advanced large language models (ChatGPT and GPT 4.0) and a deep learning-based image recognition model (Dino V2) to provide dietary recommendations and ingredient-level meal analysis. ChatGPT demonstrated proficiency by passing the Chinese Registered Dietitian Examination and generating responses that aligned well with expert recommendations, though inconsistencies were noted for certain Chinese-specific foods. The image recognition model achieved high accuracy in identifying ingredients, outperforming previous models. A user-friendly WeChat mini-program was developed to enhance patient engagement by enabling automated meal logging and dietary feedback. Despite promising results, limitations include variability in AI responses and the need for a defined question scope. The findings support advancing this AI nutritionist program to a clinical pilot study to assess its real-world effectiveness in improving patient adherence to dietary recommendations and health outcomes.

### 5.4 LLMs for data extraction

RAG-LLMs are an exciting extension of regular LLMs that make use of an external knowledge base in order to enhance the LLM’s performance. Alkhalaf and co-workers evaluated the effectiveness of using the open-source Llama 2 LLM with zero-shot prompt engineering, both alone and combined with RAG, to summarize and extract malnutrition-related data from electronic health records (EHRs) in Australian aged care facilities (65). Results showed that the model achieved high accuracy in summarizing structured malnutrition notes (93.25%) and extracting risk factors (90%), with RAG integration further improving summarization accuracy to 99.25%. While the model effectively processed explicit information, it encountered hallucination issues when details were implicit or missing. The RAG approach mitigated these limitations by providing relevant external data, enhancing the model’s ability to generate accurate, contextually relevant outputs. The findings underscore the potential of LLMs combined with RAG to streamline EHR data analysis, improve care quality, and support timely interventions for malnutrition and other healthcare challenges in aged care settings.

### 5.5 Cross-disciplinary innovations with potential for clinical nutrition

Although ChatGPT and similar LLMs can be useful, hallucinations and incomplete information are still important drawbacks. Lee et al. developed and evaluated a dual retrieval-augmented generation system to enhance the accuracy and reliability of LLMs in diabetes management across diverse languages and guidelines (66). By integrating dense and sparse retrieval methods, the system addressed limitations in semantic and keyword-based searches, utilizing dense retrievers like Solar Embedding-1-large and OpenAI’s text-embedding-3-large alongside the BM25 algorithm for sparse retrieval. Evaluation using the 2023 Korean and American diabetes guidelines demonstrated superior performance for ensemble retrievers, reducing hallucinations and maintaining high retrieval precision. The system highlights the potential for cross-regional applications, offering a scalable solution to provide accurate, current medical information in dynamic fields like diabetes management while minimizing the need for frequent LLM retraining. This strategy could be adopted in clinical nutrition for LLMs with better performance.

LLMs hold substantial potential to enhance nutritional care by supporting dietary recommendations, information and education, ingredient analysis, and data extraction. However, challenges such as limited accuracy, incomplete outputs, and hallucinations remain significant barriers to their clinical adoption. Emerging strategies like retrieval-augmented generation and fine-tuning, already being applied in other medical domains such as diabetes care, offer promising pathways to overcome these limitations (67, 68). Adapting these solutions to the nutritional context will be crucial for developing safe, reliable, and context-aware LLMs that can meaningfully support clinicians and patients alike.

## 6 Limitations and challenges of LLMs

Although LLMs have impressive potential, their capabilities are accompanied by a range of limitations and challenges that constrain their effectiveness, reliability, and ethical deployment. These challenges span technical, practical, and societal domains.

### 6.1 Data quality

One fundamental limitation of LLMs lies in their reliance on training data. These models learn patterns and relationships from vast corpora of text, but their performance is inherently constrained by the quality, diversity, and representativeness of the data used for training. Biases present in the training data are often reflected in the outputs of LLMs, perpetuating stereotypes and inequities (69). Biased representations of gender, race, or cultural norms within datasets can lead to outputs that reinforce these biases, posing ethical challenges in applications where neutrality and fairness are paramount (70–72). For example, when using an LLM to generate dietary advice, the model should be able to take into account individual factors such as cultural background, religious dietary practices, and regional food availability. Achieving this requires training data that accurately reflects diverse populations and dietary contexts. If the underlying data lacks this diversity or contains cultural biases, the model may produce dietary recommendations that are unsuitable or insensitive, potentially undermining patient trust and limiting the clinical usefulness of the advice.

### 6.2 Accuracy of LLMs

LLMs exhibit limitations in reasoning and factual accuracy (73–76). Although they are capable of generating coherent and contextually appropriate responses, their knowledge is static and limited to the data they were trained on, which often has a cutoff date. This means they cannot access or incorporate new information that arises after their training. Furthermore, LLMs lack a true understanding of the concepts they process, relying instead on probabilistic patterns to predict outputs. This can lead to hallucinations, where the model generates plausible-sounding but incorrect or nonsensical information. Such inaccuracies pose risks in critical fields like clinical nutrition and healthcare in general, where errors can have significant consequences. For instance, an LLM might incorrectly recommend micronutrient dosages that exceed safe upper limits, fail to account for specific dietary restrictions in food allergy, or generate outdated guidance on nutritional care in gestational diabetes. Inaccurate interpretation of lab values or mismatched nutritional protocols for conditions like celiac disease or refeeding syndrome could further compromise patient safety. These examples highlight that LLMs have clear limitations that nutrition specialists must be aware of and actively consider in clinical practice. They underscore the importance of rigorous human oversight, domain-specific fine-tuning, and real-time validation when applying LLMs in clinical decision-making.

### 6.3 Computational and energy costs

Another significant challenge is the computational intensity of LLMs. Training and deploying these models requires substantial computational resources, including high-performance hardware and significant energy consumption (77). This raises concerns about the environmental impact of large-scale LLMs, as well as their accessibility to smaller organizations or institutions with limited resources. The high cost associated with developing and maintaining these models exacerbates the divide between well-funded entities and smaller players, potentially centralizing control of this transformative technology. This economic barrier has direct implications for nutritional care in low-resource countries, where the burden of undernutrition is high, access to trained dietitians is limited, and current clinical guidelines are often unavailable. In these settings, the potential value of LLMs may be even greater. Yet the high cost of implementing such tools risks placing them out of reach precisely where they could make the most impact. Without targeted strategies to improve accessibility, the use of LLMs in clinical nutrition may inadvertently deepen global health disparities rather than help to close them.

### 6.4 Transparency

LLMs also face challenges in interpretability and explainability (78). Despite their remarkable capabilities, the decision-making processes of these models are often opaque, making it difficult to understand why a particular output was generated. This lack of transparency complicates their integration into domains requiring accountability, such as clinical nutrition. In these contexts, stakeholders need to trust the system’s outputs and have mechanisms to verify or challenge its conclusions, yet the black-box nature of LLMs undermines this trust. In clinical nutrition, transparency is especially important. Dietitians and nutrition physicians must be able to explain the rationale behind their recommendations, whether for an individualized dietary plan, a nutrient prescription, or a nutritional intervention for a complex patient. If an LLM suggests a course of action, clinicians must be able to assess where that advice came from and whether it aligns with current guidelines and the patient’s specific context.

Without a clear link between input, reasoning, and output, clinicians are left in a difficult position: either they accept the model’s advice without understanding its basis, or they disregard it altogether. Neither approach supports responsible, evidence-based care. For LLMs to be meaningfully integrated into clinical nutrition, they must offer more than just plausible suggestions; they must provide traceable reasoning, cite their sources where possible, and allow users to interrogate the path that led to a given recommendation. Transparency is not just a technical concern; it is central to clinical responsibility, professional credibility, and patient safety. Without it, the promise of LLMs in nutrition remains incomplete.

### 6.5 Contextual awareness

The models’ inability to handle contextual nuances and ambiguities effectively further limits their utility. While LLMs excel at generating text based on syntactic and semantic patterns, they may struggle to interpret subtle nuances, sarcasm, idiomatic expressions, or culturally specific references (79). This limitation becomes particularly problematic in multilingual or cross-cultural applications, where the model’s understanding of context may diverge significantly from human expectations. In clinical nutrition, where patient communication is central and often nuanced, this lack of contextual awareness can be a serious concern. For instance, a phrase like “I eat light” or “I do not eat much during the day” can carry very different meanings depending on the person’s background, culture, or even local habits. Without an understanding of that context, a model may misread the intent entirely. The same goes for dietary preferences or restrictions, which are sometimes expressed in everyday or non-standard language. This is particularly true in multilingual settings, where nuance is easily lost. Understanding what a patient really means often requires not just linguistic knowledge but also cultural sensitivity and clinical experience.

### 6.6 Expert knowledge

LLMs often lack domain-specific expertise, particularly when applied to specialized fields without additional fine-tuning or context augmentation (80). Their generalized training enables them to perform adequately across a broad range of tasks but often fails to meet the rigor and precision required in highly technical areas. Without domain adaptation, their outputs risk being overly generic, superficial, or inaccurate in professional settings. In clinical nutrition, recommendations must be tailored not only to individual patient needs but also to complex physiological conditions, disease states, and evidence-based guidelines. In more challenging scenarios, such as managing refeeding syndrome or formulating parenteral nutrition plans, superficial suggestions from a general-purpose LLM could mislead rather than support the clinician. To be truly useful in clinical nutrition, LLMs need more than fluent language; they require structured exposure to clinical nutrition guidelines from authoritative nutrition sources.

### 6.7 Human oversight

Finally, user interactions with LLMs present challenges related to over-reliance and the need for human oversight. Because LLMs generate text that appears authoritative and well-informed, users may overestimate their reliability, failing to scrutinize outputs critically (81). This can lead to erroneous decisions, particularly in high-stakes environments where unchecked reliance on model outputs can have severe repercussions. Therefore, human oversight remains imperative.

While LLMs represent a significant advancement in artificial intelligence, their limitations and challenges, as discussed in this section, underscore the need for continued research and thoughtful deployment. Addressing issues such as data bias, factual accuracy, computational demands, interpretability, and ethical safeguards is critical to ensure that these models are used responsibly and equitably. By acknowledging and addressing these challenges, the field can harness the transformative potential of LLMs while mitigating the risks they pose.

## 7 Future directions of LLMs in clinical nutrition

The future of LLMs in clinical nutrition holds immense potential to transform the field by enabling more personalized, evidence-based, and scalable approaches to patient care. As advancements in artificial intelligence continue to unfold, LLMs are poised to play a pivotal role in integrating complex nutritional data, facilitating decision-making, and empowering healthcare professionals to address the growing burden of nutrition-related diseases (82, 83).

One of the most promising applications of LLMs in clinical nutrition is their ability to synthesize vast amounts of nutritional and medical data to support personalized dietary recommendations (84–86). Nutrition is highly individualized, influenced by factors such as age, sex, genetics, metabolic profile, lifestyle, and comorbidities. LLMs, when integrated with data from wearable devices, EHRs, and genetic testing, could analyze these diverse inputs to generate tailored dietary plans. For instance, an LLM could consider a patient’s metabolic panel, body composition analysis, and physical activity data to recommend precise macronutrient and micronutrient targets, addressing specific health goals such as weight management, glycemic control, or reducing cardiovascular risk.

The integration of LLMs with predictive analytics and machine learning models could further enhance their utility in clinical nutrition (87). By analyzing longitudinal health data, LLMs could predict an individual’s risk of developing nutrition-related conditions, such as type 2 diabetes or cardiovascular disease, and recommend preemptive dietary interventions. This proactive approach aligns with the broader goals of preventive medicine, shifting the focus from treating disease to maintaining health and wellness.

LLMs could also address challenges related to cultural and linguistic diversity in clinical nutrition. Nutrition advice must often be adapted to cultural preferences, traditional cuisines, and local food availability. LLMs trained on multilingual and culturally diverse datasets could assist healthcare providers in delivering culturally sensitive dietary recommendations. For example, an LLM could help tailor meal plans for a patient while taking into consideration specific dietary restrictions due to religious practices or cultural norms, ensuring greater relevance and acceptability of the guidance provided.

Another key area for the future application of LLMs in clinical nutrition is patient education and engagement (88–90). LLMs excel at generating human-like text, making them ideal tools for creating patient-facing educational materials, answering frequently asked questions, and providing real-time support (91). For example, an LLM-powered chatbot could assist patients in understanding dietary restrictions, decoding food labels, or identifying suitable recipes that align with their medical conditions and personal preferences. By delivering accessible and contextually relevant information, these models can empower patients to make informed decisions about their nutrition, fostering adherence to prescribed dietary regimens.

In the realm of research, LLMs could serve as invaluable tools for evidence synthesis and knowledge translation in clinical nutrition (92, 93). The volume of published nutritional science research grows rapidly, making it challenging for practitioners to stay current. LLMs can summarize recent studies, identify emerging trends, and highlight consensus or controversies within the field. Furthermore, these models could aid researchers in generating hypotheses by identifying gaps in the literature, fostering innovation in nutritional science.

Collaboration between healthcare providers, data scientists, and policymakers will be crucial in shaping the future of LLMs in clinical nutrition. Developing standardized protocols for integrating LLMs into clinical workflows and establishing guidelines for their ethical use will be vital steps in realizing their potential. Moreover, ongoing education and training for clinicians on the capabilities and limitations of LLMs will empower them to harness these tools effectively while maintaining critical oversight (94, 95).

LLMs offer transformative possibilities for advancing personalized, preventive, and culturally sensitive approaches in clinical nutrition. Realizing this potential will depend on thoughtful integration into clinical practice, guided by interdisciplinary collaboration, ethical oversight, and clinician education.

## 8 Discussion

Large language models offer considerable promise for advancing clinical nutrition. Their ability to interpret complex data, generate tailored dietary recommendations, and assist both clinicians and patients in decision-making aligns with the increasing need for scalable, personalized, and evidence-based care. Yet, despite their potential, the practical and ethical integration of LLMs into clinical nutrition remains a complex undertaking.

One of the primary concerns is the reliability of LLM-generated outputs. Although these models often produce coherent and convincing responses, they remain vulnerable to factual inaccuracies and hallucinations. This is a pressing issue in clinical contexts where incorrect information can have significant consequences. Mistakes in nutrient recommendations, dietary restrictions, or the management of nutrition-related conditions could compromise patient safety. As such, validation procedures, domain-specific fine-tuning, and routine human oversight are critical to ensure these tools support rather than undermine clinical judgment.

Ethical challenges further complicate implementation. LLMs are shaped by the data on which they are trained, and if that data lacks cultural, linguistic, or socioeconomic diversity, the outputs may inadvertently reinforce existing disparities. For example, a model might fail to account for local dietary practices or regional food availability, reducing the relevance and acceptability of its recommendations. Developing and training LLMs on more inclusive, representative datasets is essential to making their outputs both equitable and clinically useful.

Another key factor is the integration of LLMs into existing healthcare systems. To deliver real value, these tools must interact seamlessly with electronic health records, clinical decision support systems, and other digital health infrastructure. Achieving this requires not only technical interoperability but also regulatory alignment and user training. Without these elements in place, LLMs risk becoming isolated solutions that fail to improve efficiency or care quality in practice.

Equally important is the perception of both clinicians and patients. While many recognize the potential of AI tools in healthcare, concerns persist about the transparency, trustworthiness, and impersonal nature of automated advice. Designing user interfaces that are clear, interactive, and adaptable to individual preferences can help address these concerns. Importantly, LLMs should be positioned as support tools that enhance rather than replace human clinical expertise.

Scalability also remains a barrier to wide-scale adoption. The substantial computational and energy requirements of LLMs can make their deployment costly and technically demanding. This is particularly problematic in low income countries, where malnutrition is highly prevalent, but the infrastructure to support AI tools is limited. When developing LLM-based tools for clinical nutrition, these issues should be taken into consideration.

Despite these limitations, LLMs represent a powerful new tool for enhancing nutrition care. They can assist in automating routine tasks, lowering barriers to dietary counseling, and expanding the availability of up-to-date, evidence-based information. Their effectiveness, however, will depend on thoughtful implementation guided by clinical priorities, ethical standards, and interdisciplinary collaboration.

In conclusion, the integration of LLMs into clinical nutrition holds considerable potential, but this promise can only be realized through deliberate, responsible development. Advances in techniques such as fine-tuning, retrieval-augmented generation, and multimodal input are improving the relevance and safety of these models. Moving forward, success will require more than technical refinement; it will demand sustained efforts to ensure that LLMs are accurate, fair, transparent, and aligned with the realities of clinical care. With appropriate safeguards and collaboration across disciplines, LLMs may ultimately become valuable allies in delivering high-quality, personalized nutrition care.

## References

[1] Bond A Mccay K Lal S. Artificial intelligence & clinical nutrition: what the future might have in store. Clin Nutr ESPEN. (2023) 57:542–9. doi: 10.1016/j.clnesp.2023.07.082, PMID: 37739704

[2] Belkhouribchia J. Artificial intelligence is going to transform the field of endocrinology: an overview. Front Endocrinol (Lausanne). (2025) 16:1513929. doi: 10.3389/fendo.2025.1513929, PMID: 39882100 PMC11772191

[3] Singhal K Azizi S Tu T Mahdavi SS Wei J Chung HW. Large language models encode clinical knowledge. Nature. (2023) 620:172–80. doi: 10.1038/s41586-023-06291-2, PMID: 37438534 PMC10396962

[4] Ogrinc M Koroušić Seljak B Eftimov T. Zero-shot evaluation of ChatGPT for food named-entity recognition and linking. Front Nutr. (2024) 11:1429259. doi: 10.3389/fnut.2024.1429259, PMID: 39290564 PMC11406469

[5] Iqbal U Tanweer A Rahmanti AR Greenfield D Lee LT Li YJ. Impact of large language model (ChatGPT) in healthcare: an umbrella review and evidence synthesis. J Biomed Sci. (2025) 32:45. doi: 10.1186/s12929-025-01131-z, PMID: 40335969 PMC12057020

[6] Yu E Chu X Zhang W Meng X Yang Y Ji X. Large language models in medicine: applications, challenges, and future directions. Int J Med Sci. (2025) 22:2792–801. doi: 10.7150/ijms.111780, PMID: 40520893 PMC12163604

[7] Su H Sun Y Li R Zhang A Yang Y Xiao F. Large language models in medical diagnostics: scoping review with bibliometric analysis. J Med Internet Res. (2025) 27:e72062. doi: 10.2196/72062, PMID: 40489764 PMC12186007

[8] Qin H Tong Y. Opportunities and challenges for large language models in primary health care. J Prim Care Community Health. (2025) 16:21501319241312571. doi: 10.1177/21501319241312571, PMID: 40162893 PMC11960148

[9] Preiksaitis C Ashenburg N Bunney G Chu A Kabeer R Riley F. The role of large language models in transforming emergency medicine: scoping review. JMIR Med Inform. (2024) 12:e53787. doi: 10.2196/53787, PMID: 38728687 PMC11127144

[10] Thirunavukarasu AJ Ting DSJ Elangovan K Gutierrez L Tan TF Ting DSW. Large language models in medicine. Nat Med. (2023) 29:1930–40. doi: 10.1038/s41591-023-02448-8, PMID: 37460753

[11] Dergaa I Chamari K Zmijewski P Ben Saad H. From human writing to artificial intelligence generated text: examining the prospects and potential threats of ChatGPT in academic writing. Biol Sport. (2023) 40:615–22. doi: 10.5114/biolsport.2023.125623, PMID: 37077800 PMC10108763

[12] Bergling K Wang LC Shivakumar O Nandorine Ban A Moore LW Ginsberg N. From bytes to bites: application of large language models to enhance nutritional recommendations. Clin Kidney J. (2025) 18:sfaf 082. doi: 10.1093/ckj/sfaf082, PMID: 40226366 PMC11992566

[13] Busch F Hoffmann L Rueger C van Dijk EH Kader R Ortiz-Prado E. Current applications and challenges in large language models for patient care: a systematic review. Commun Med (Lond). (2025) 5:26. doi: 10.1038/s43856-024-00717-2, PMID: 39838160 PMC11751060

[14] Kim J Vajravelu BN. Assessing the current limitations of large language models in advancing health care education. JMIR Form Res. (2025) 9:e51319. doi: 10.2196/51319, PMID: 39819585 PMC11756841

[15] Wang L Wan Z Ni C Song Q Li Y Clayton E. Applications and concerns of ChatGPT and other conversational large language models in health care: systematic review. J Med Internet Res. (2024) 26:e22769. doi: 10.2196/22769, PMID: 39509695 PMC11582494

[16] Girouard MP Chang AJ Liang Y Hamilton SA Bhatt AS Svetlichnaya J. Clinical and research applications of natural language processing for heart failure. Heart Fail Rev. (2024) 30:407–15. doi: 10.1007/s10741-024-10472-0, PMID: 39699708

[17] Yang X Li T Su Q Liu Y Kang C Lyu Y. Application of large language models in disease diagnosis and treatment. Chin Med J. (2025) 138:130–42. doi: 10.1097/CM9.0000000000003456, PMID: 39722188 PMC11745858

[18] Lee J Park S Shin J Cho B. Analyzing evaluation methods for large language models in the medical field: a scoping review. BMC Med Inform Decis Mak. (2024) 24:366. doi: 10.1186/s12911-024-02709-7, PMID: 39614219 PMC11606129

[19] Wong IN Monteiro O Baptista-Hon DT Wang K Lu W Sun Z. Leveraging foundation and large language models in medical artificial intelligence. Chin Med J. (2024) 137:2529–39. doi: 10.1097/CM9.0000000000003302, PMID: 39497256 PMC11556979

[20] Vaswani A Shazeer N Parmar N Uszkoreit J Jones L Gomez AN. Attention is all you need. Advances in neural information processing systems 30: annual conference on neural information processing systems 2017. Long Beach, CA, USA: Curran Associates, Inc. (2017):5998–6008. doi: 10.48550/arXiv.1706.03762

[21] Denecke K May R Rivera-Romero O. Transformer models in healthcare: a survey and thematic analysis of potentials, shortcomings and risks. J Med Syst. (2024) 20:23. doi: 10.1007/s10916-024-02043-5, PMID: 38367119 PMC10874304

[22] Madan S Lentzen M Brandt J Rueckert D Hofmann-Apitius M Fröhlich H. Transformer models in biomedicine. BMC Med Inform Decis Mak. (2024) 24:214. doi: 10.1186/s12911-024-02600-5, PMID: 39075407 PMC11287876

[23] Dotan E Jaschek G Pupko T Belinkov Y. Effect of tokenization on transformers for biological sequences. Bioinformatics. (2024) 40:btae196. doi: 10.1093/bioinformatics/btae196, PMID: 38608190 PMC11055402

[24] Zhang C Peng B Sun X Niu Q Liu J Chen K. From word vectors to multimodal embeddings: techniques, applications, and future directions for large language models. ar Xiv. (2024). doi: 10.48550/arXiv.2411.05036

[25] Bergmann D Stryker C. (2024) What is an attention mechanism? IBM. Available online at: https://www.ibm.com/think/topics/attention-mechanism (Accessed on 2025 Jan 30

[26] Ye L Tao Z Huang Y Li Y. Chunkattention: efficient self-attention with prefix-aware KV cache and two-phase partition. arXiv. (2024). doi: 10.48550/arXiv.2402.15220

[27] Amatriain X Sankar A Bing J Bodigutla PK Hazen TJ Kazi M. (2023). Transformer models: an introduction and catalog. arXiv. doi: 10.48550/arXiv.2302.07730

[28] Rashno E Eskandari A Anand A Zulkernine F. (2024). Survey: transformer-based models in data modality conversion. arXiv. doi: 10.48550/arXiv.2408.04723

[29] Devlin J Chang MW Lee K Toutanova K. BERT: pre-training of deep bidirectional transformers for language understanding. arXiv. (2018) :1810.04805. doi: 10.48550/arXiv.1810.04805

[30] Roberts J “How powerful are decoder-only transformer neural models?” In: *Proceedings of the international joint conference on neural networks (IJCNN)*; (2024).

[31] Kementchedjhieva Y Chalkidis I. An exploration of encoder-decoder approaches to multi-label classification for legal and biomedical text. arXiv. (2023). doi: 10.48550/arXiv.2305.05627

[32] Raschka S. Understanding Encoder and Decoder LLMs. Ahead of AI. (2023)

[33] Hou Y Bishop JR Liu H Zhang R. Improving dietary supplement information retrieval: development of a retrieval-augmented generation system with large language models. J Med Internet Res. (2025) 27:e67677. doi: 10.2196/67677, PMID: 40106799 PMC11966073

[34] Azimi I Qi M Wang L Rahmani AM Li Y. Evaluation of LLMs accuracy and consistency in the registered dietitian exam through prompt engineering and knowledge retrieval. Sci Rep. (2025) 15:1506. doi: 10.1038/s41598-024-85003-w, PMID: 39789057 PMC11718202

[35] Zarecki I. RAG vs fine-tuning vs prompt engineering: and the winner is…. K2View; (2024). Available online at: https://www.k2view.com/blog/rag-vs-fine-tuning-vs-prompt-engineering/#Prompt-engineering-is-an-essential-RAG-component (Accessed on 2025 Jan 30)

[36] Belcic I Stryker C. RAG vs. fine-tuning. IBM. (2024)

[37] Meskó B. Prompt engineering as an important emerging skill for medical professionals: tutorial. J Med Internet Res. (2023) 25:e50638. doi: 10.2196/50638, PMID: 37792434 PMC10585440

[38] White J Fu Q Hays S Sandborn M Olea C Gilbert H. A prompt pattern catalog to enhance prompt engineering with ChatGPT. arXiv. (2023):11382. doi: 10.48550/arXiv.2302.11382

[39] Kim TT Makutonin M Sirous R Javan R. Optimizing large language models in radiology and mitigating pitfalls: prompt engineering and fine-tuning. Radiographics. (2025) 45:e240073. doi: 10.1148/rg.240073, PMID: 40048389

[40] Oniani D Wu X Visweswaran S Kapoor S Kooragayalu S Polanska K. Enhancing large language models for clinical decision support by incorporating clinical practice guidelines. Proc (IEEE Int Conf Healthc Inform). (2024) 2024:694–702. doi: 10.1109/ichi61247.2024.00111 40092288 PMC11909794

[41] Bürgisser N Chalot E Mehouachi S Buclin CP Lauper K Courvoisier DS. Large language models for accurate disease detection in electronic health records: the examples of crystal arthropathies. RMD Open. (2024) 10:e005003. doi: 10.1136/rmdopen-2024-005003, PMID: 39794274 PMC11664341

[42] Wrightson JG Blazey P Moher D Khan KM Ardern CL. GPT for RCTs? Using AI to determine adherence to clinical trial reporting guidelines. BMJ Open. (2025) 15:e088735. doi: 10.1136/bmjopen-2024-088735, PMID: 40107689 PMC11927406

[43] Rangan K Yin Y. A fine-tuning enhanced RAG system with quantized influence measure as AI judge. Sci Rep. (2024) 14:27446. doi: 10.1038/s41598-024-79110-x, PMID: 39523408 PMC11551171

[44] Tozuka R Johno H Amakawa A Sato J Muto M Seki S. Application of notebook LM, a large language model with retrieval-augmented generation, for lung cancer staging. Jpn J Radiol. (2025) 43:706–12. doi: 10.1007/s11604-024-01705-1, PMID: 39585559

[45] Vrdoljak J Boban Z Vilović M Kumrić M Božić J. A review of large language models in medical education, clinical decision support, and healthcare administration. Healthcare (Basel). (2025) 13:603. doi: 10.3390/healthcare13060603, PMID: 40150453 PMC11942098

[46] Meskó B. The impact of multimodal large language models on health care's future. J Med Internet Res. (2023) 25:e52865. doi: 10.2196/52865, PMID: 37917126 PMC10654899

[47] Bochtler M. How the technologies behind self-driving cars, social networks, ChatGPT, and DALL-E2 are changing structural biology. BioEssays. (2025) 47:e2400155. doi: 10.1002/bies.202400155, PMID: 39404756 PMC11662154

[48] Chen J Wu X Lan T Li B. LLMER: crafting interactive extended reality worlds with JSON data generated by large language models. IEEE Trans Vis Comput Graph. (2025) 31:2715–24. doi: 10.1109/TVCG.2025.3549549, PMID: 40063485

[49] Li J Guan Z Wang J Cheung CY Zheng Y Lim LL. Integrated image-based deep learning and language models for primary diabetes care. Nat Med. (2024) 30:2886–96. doi: 10.1038/s41591-024-03139-8, PMID: 39030266 PMC11485246

[50] Singh B Olds T Brinsley J Dumuid D Virgara R Matricciani L. Systematic review and meta-analysis of the effectiveness of chatbots on lifestyle behaviours. NPJ Digit Med. (2023) 6:118. doi: 10.1038/s41746-023-00856-1, PMID: 37353578 PMC10290125

[51] Arslan S. Exploring the potential of chat GPT in personalized obesity treatment. Ann Biomed Eng. (2023) 51:1887–8. doi: 10.1007/s10439-023-03227-9, PMID: 37145177

[52] Haman M Školník M Lošťák M. AI dietician: unveiling the accuracy of ChatGPT's nutritional estimations. Nutrition. (2024) 119:112325. doi: 10.1016/j.nut.2023.112325, PMID: 38194819

[53] Khan U. Revolutionizing personalized protein energy malnutrition treatment: harnessing the power of chat GPT. Ann Biomed Eng. (2024) 52:1125–7. doi: 10.1007/s10439-023-03331-w, PMID: 37728811

[54] Wang LC Zhang H Ginsberg N Nandorine Ban A Kooman JP Kotanko P. Application of ChatGPT to support nutritional recommendations for Dialysis patients - a qualitative and quantitative evaluation. J Ren Nutr. (2024) 34:477–81. doi: 10.1053/j.jrn.2024.09.001, PMID: 39278578

[55] Adilmetova G Nassyrov R Meyerbekova A Karabay A Varol HA Chan MY. Evaluating ChatGPT's multilingual performance in clinical nutrition advice using synthetic medical text: insights from Central Asia. J Nutr. (2024) 155:729–735. doi: 10.1016/j.tjnut.2024.12.018 39732434

[56] Hieronimus B Hammann S Podszun MC. Can the AI tools ChatGPT and bard generate energy, macro- and micro-nutrient sufficient meal plans for different dietary patterns? Nutr Res. (2024) 128:105–14. doi: 10.1016/j.nutres.2024.07.002, PMID: 39102765

[57] Niszczota P Rybicka I. The credibility of dietary advice formulated by ChatGPT: Robo-diets for people with food allergies. Nutrition. (2023) 112:112076. doi: 10.1016/j.nut.2023.112076, PMID: 37269717

[58] Papastratis I Konstantinidis D Daras P Dimitropoulos K. AI nutrition recommendation using a deep generative model and ChatGPT. Sci Rep. (2024) 14:14620. doi: 10.1038/s41598-024-65438-x, PMID: 38918477 PMC11199627

[59] Barlas T Altinova AE Akturk M Toruner FB. Credibility of ChatGPT in the assessment of obesity in type 2 diabetes according to the guidelines. Int J Obes. (2024) 48:271–5. doi: 10.1038/s41366-023-01410-5, PMID: 37951982

[60] Van Delanotte C Hodgetts D Peris DLIHK Karki A Maher C Imam T. Perceptions and expectations of an artificially intelligent physical activity digital assistant - a focus group study. Appl Psychol Health Well-Being. (2024) 16:2362–80. doi: 10.1111/aphw.12594 39268568

[61] Kirk D van Eijnatten E Camps G. Comparison of answers between ChatGPT and human dieticians to common nutrition questions. J Nutr Metab. (2023) 2023:1–9. doi: 10.1155/2023/5548684, PMID: 38025546 PMC10645493

[62] Liao LL Chang LC Lai IJ. Assessing the quality of ChatGPT's dietary advice for college students from dietitians' perspectives. Nutrients. (2024) 16:1939. doi: 10.3390/nu16121939, PMID: 38931294 PMC11206595

[63] Hoang YN Chen YL Ho DKN Chiu WC Cheah KJ Mayasari NR. Consistency and accuracy of artificial intelligence for providing nutritional information. JAMA Netw Open. (2023) 6:e2350367. doi: 10.1001/jamanetworkopen.2023.50367, PMID: 38150258 PMC10753390

[64] Sun H Zhang K Lan W Gu Q Jiang G Yang X. An AI dietitian for type 2 diabetes mellitus management based on large language and image recognition models: preclinical concept validation study. J Med Internet Res. (2023) 25:e51300. doi: 10.2196/51300, PMID: 37943581 PMC10667983

[65] Alkhalaf M Yu P Yin M Deng C. Applying generative AI with retrieval augmented generation to summarize and extract key clinical information from electronic health records. J Biomed Inform. (2024) 156:104662. doi: 10.1016/j.jbi.2024.104662 38880236

[66] Lee J Cha H Hwangbo Y Cheon W. Enhancing large language model reliability: minimizing hallucinations with dual retrieval-augmented generation based on the latest diabetes guidelines. J Pers Med. (2024) 14:1131. doi: 10.3390/jpm14121131, PMID: 39728044 PMC11677479

[67] Li M Kilicoglu H Xu H Zhang R. Biomed RAG: a retrieval augmented large language model for biomedicine. J Biomed Inform. (2025) 162:104769. doi: 10.1016/j.jbi.2024.104769, PMID: 39814274 PMC11837810

[68] Sonnenburg A van der Lugt B Rehn J Wittkowski P Bech K Padberg F. Artificial intelligence-based data extraction for next generation risk assessment: is fine-tuning of a large language model worth the effort? Toxicology. (2024) 508:153933. doi: 10.1016/j.tox.2024.153933, PMID: 39181527

[69] Clusmann J Kolbinger FR Muti HS Carrero ZI Eckardt JN Laleh NG. The future landscape of large language models in medicine. Commun Med (Lond). (2023) 3:141. doi: 10.1038/s43856-023-00370-1, PMID: 37816837 PMC10564921

[70] Hilling DE Ihaddouchen I Buijsman S Townsend R Gommers D van Genderen ME. The imperative of diversity and equity for the adoption of responsible AI in healthcare. Front Artif Intell. (2025) 8:1577529. doi: 10.3389/frai.2025.1577529, PMID: 40309720 PMC12040885

[71] Ayoub NF Balakrishnan K Ayoub MS Barrett TF David AP Gray ST. Inherent Bias in large language models: a random sampling analysis. Mayo Clin Proc Digit Health. (2024) 2:186–91. doi: 10.1016/j.mcpdig.2024.03.003, PMID: 40207170 PMC11975844

[72] Schnepper R Roemmel N Schaefert R Lambrecht-Walzinger L Meinlschmidt G. Exploring biases of large language models in the field of mental health: comparative questionnaire study of the effect of gender and sexual orientation in anorexia nervosa and bulimia nervosa case vignettes. JMIR Ment Health. (2025) 12:e57986. doi: 10.2196/57986, PMID: 40111287 PMC11949086

[73] Liang S Zhang J Liu X Huang Y Shao J Liu X. The potential of large language models to advance precision oncology. EBioMedicine. (2025) 115:105695. doi: 10.1016/j.ebiom.2025.105695, PMID: 40305985 PMC12083916

[74] Chen D Parsa R Swanson K Nunez JJ Critch A Bitterman DS. Large language models in oncology: a review. BMJ Oncol. (2025) 4:e000759. doi: 10.1136/bmjonc-2025-000759, PMID: 40519217 PMC12164365

[75] Chen D Avison K Alnassar S Huang RS Raman S. Medical accuracy of artificial intelligence chatbots in oncology: a scoping review. Oncologist. (2025) 30:oyaf 038. doi: 10.1093/oncolo/oyaf038, PMID: 40285677 PMC12032582

[76] Biesheuvel LA Workum JD Reuland M van Genderen ME Thoral P Dongelmans D. Large language models in critical care. J Intensive Med. (2024) 5:113–8. doi: 10.1016/j.jointm.2024.12.001 40241839 PMC11997603

[77] Rybinski M Kusa W Karimi S Hanbury A. Learning to match patients to clinical trials using large language models. J Biomed Inform. (2024) 159:104734. doi: 10.1016/j.jbi.2024.104734, PMID: 39389283

[78] Berkowitz J Weissenbacher D Srinivasan A Friedrich NA Acitores Cortina JM Kivelson S. Probing large language model hidden states for adverse drug reaction knowledge. med Rxiv. (2025) 2025:25321620. doi: 10.1101/2025.02.09.25321620

[79] Chen CC Chen JA Liang CS Lin YH. Large language models may struggle to detect culturally embedded filicide-suicide risks. Asian J Psychiatr. (2025) 105:104395. doi: 10.1016/j.ajp.2025.104395, PMID: 39955914

[80] Bicknell BT Rivers NJ Skelton A Sheehan D Hodges C Fairburn SC. Domain-specific customization for language models in otolaryngology: the ENT GPT assistant. OTO Open. (2025) 9:e70125. doi: 10.1002/oto2.70125, PMID: 40331108 PMC12051367

[81] Singh S Chaurasia A Raichandani S Grewal H Udare A Jawahar A. Commentary: leveraging large language models for radiology education and training. J Comput Assist Tomogr. (2025). doi: 10.1097/RCT.0000000000001736, PMID: 40164970

[82] Iyer R Christie AP Madhavapeddy A Reynolds S Sutherland W Jaffer S. Careful design of large language model pipelines enables expert-level retrieval of evidence-based information from syntheses and databases. PLoS One. (2025) 20:e0323563. doi: 10.1371/journal.pone.0323563, PMID: 40373077 PMC12080840

[83] Ganzinger M Kunz N Fuchs P Lyu CK Loos M Dugas M. Automated generation of discharge summaries: leveraging large language models with clinical data. Sci Rep. (2025) 15:16466. doi: 10.1038/s41598-025-01618-7, PMID: 40355506 PMC12069548

[84] Omar M Nadkarni GN Klang E Glicksberg BS. Large language models in medicine: a review of current clinical trials across healthcare applications. PLOS Digit Health. (2024) 3:e0000662. doi: 10.1371/journal.pdig.0000662, PMID: 39561120 PMC11575759

[85] Mu Y He D. The potential applications and challenges of ChatGPT in the medical field. Int J Gen Med. (2024) 17:817–26. doi: 10.2147/IJGM.S456659, PMID: 38476626 PMC10929156

[86] Logan JA Sadhu S Hazlewood C Denton M Burke SE Simone-Soule CA. Bridging gaps in Cancer care: utilizing large language models for accessible dietary recommendations. Nutrients. (2025) 17:1176. doi: 10.3390/nu17071176, PMID: 40218934 PMC11990115

[87] Phalle A Gokhale D. Navigating next-gen nutrition care using artificial intelligence-assisted dietary assessment tools-a scoping review of potential applications. Front Nutr. (2025) 12:1518466. doi: 10.3389/fnut.2025.1518466, PMID: 39917741 PMC11798783

[88] LinCKuoCF. Roles and potential of large language models in healthcare: a comprehensive review. Biom J. (2025):100868. doi: 10.1016/j.bj.2025.100868, PMID: 40311872

[89] AlSammarraie A Househ M. The use of large language models in generating patient education materials: a scoping review. Acta Inform Med. (2025) 33:4–10. doi: 10.5455/aim.2024.33.4-10, PMID: 40223858 PMC11986337

[90] Yan Z Liu J Fan Y Lu S Xu D Yang Y. Ability of ChatGPT to replace doctors in patient education: cross-sectional comparative analysis of inflammatory bowel disease. J Med Internet Res. (2025) 27:e62857. doi: 10.2196/62857, PMID: 40163853 PMC11997527

[91] Rahmanti AR Yang HC Bintoro BS Nursetyo AA Muhtar MS Syed-Abdul S. Slim me, a chatbot with artificial empathy for personal weight management: system design and finding. Front Nutr. (2022) 9:870775. doi: 10.3389/fnut.2022.870775 35811989 PMC9260382

[92] Wang L Li J Zhuang B Huang S Fang M Wang C. Accuracy of large language models when answering clinical research questions: systematic review and network Meta-analysis. J Med Internet Res. (2025) 27:e64486. doi: 10.2196/64486, PMID: 40305085 PMC12079073

[93] Zeng H Yin C Chai C Wang Y Dai Q Sun H. Cancer gene identification through integrating causal prompting large language model with omics data-driven causal inference. Brief Bioinform. (2025) 26:bbaf113. doi: 10.1093/bib/bbaf113, PMID: 40072848 PMC11899576

[94] Mesko B. The ChatGPT (generative artificial intelligence) revolution has made artificial intelligence approachable for medical professionals. J Med Internet Res. (2023) 25:e48392. doi: 10.2196/48392, PMID: 37347508 PMC10337400

[95] Shool S Adimi S Saboori Amleshi R Bitaraf E Golpira R Tara M. A systematic review of large language model (LLM) evaluations in clinical medicine. BMC Med Inform Decis Mak. (2025) 25:117. doi: 10.1186/s12911-025-02954-4, PMID: 40055694 PMC11889796

